# Our Vienna Letter

**Published:** 1891-07

**Authors:** 


### Correspondence.

### OUR VIENNA LETTER.

SIGHT-SEEING IN THE MECCA—GLIMPSE OF THE MEDICAL SCHOOLS—A VISIT TO LEITER'S—THE INEVITABLE CANE AND BOULANGER—DRIFTING FROM THE SUBJECT BY GASLIGHT—THE STUDENT "IS IN IT"—LIFE STUDY—DR. FINGER'S EXCELLENT WORK AND INTERESTING CLINICS—DIAGNOSIS OF GENITO-URINARY DISEASES.

II.

VIENNA, June 2, 1891.

*To The Editor :*

DEAR SIR:—After a considerable amount of sight-seeing, which seems to be a necessary part of (the business) a trip over here, I have come to a stand in this very attractive and absorbing burg—the Mecca of the medical profession, as it is often, and very justly, termed. This is indeed a haven, to which all of our kind may come with the expectation of finding most valuable opportunities for pursuing with profit the study of any branch of our much specialized art; for specialties of the most erratic nature and modern construction find places in the catalogue of the "Facultat."

The University, the Krankenhaus (general city hospital) and the Poliklinik are all located in one neighborhood, and they look like veritable bee hives, with students, teachers and patients for their busy occupants. The dimensions and capacity of these buildings must not be measured or compared with anything in our own country; I have seen nothing like it there. The Krankenhaus alone is large enough to contain a small town. A new comer can easily get lost in wandering through its many "hofs" or courts, or its endless wards. I started with a fellow student to walk around it on the outside the other evening, on our way to Leiter's (of cystoscopic fame). Well, I shall not endeavor to guess the distance covered in getting there, as my guess would seem preposperous, but I will say that we were late getting back to supper in consequence of adhering to our determination to go around the Krankenhaus.

The utmost freedom is allowed evey body in going in or out of this great institution. The courts are beautifully shaded with trees, which overhang the many well kept walks; and along these the convalescent patients, male and female, walk, chat, flirt or doze, presenting a peculiar and interesting spectacle with their numerous bandages, their splints and hospital garb as mark of distinction from the clusters of students that loll around on the benches and lend picturesqueness to the scene. But the students themselves carry a badge of distinction that serves to stamp them as such with unerring certainty; a cane is their unfailing companion. I have yet to see a medical student, who has been here long enough to become acclimated, venture out of doors without his cane! And it is also surprising to observe with what rapidity, after his arrival here, an American student becomes ingrafted with a beard—at first a scaly looking affair, but later, thanks to the solicitous attention bestowed on it by its possessor, a very respectable appearing, closely cropped Boulanger is evolved.

The life of a student of medicine here is an ideal one, and emphasizes the difference in the amount of pleasure to be derived from the *study* of medicine, as compared with its practice. Passing their days in listening to the discourses of master medical minds—recognized leaders of the profession; watching the clinics, which afford examples of disease, of all possible varieties; delving in laboratory work, original and otherwise; viewing operations by masters of the surgical art; with an interval—a remission from such arduous tasks—of from one to three hours at noon, to eat, chat, smoke or read in the cafes. But at night is the time when the busy medical student is allowed the liberty of relaxation and enjoyment. And where in the world are the opportunities for obtaining these better than in Vienna, the very cradle of delicious music, of beer, of dazzling frauliens, an irrrsistible combination, all of which is to be found "im Prater" and in the concert gardens?

All Vienna goes to these resorts nightly, and why should not the poor, over-worked medical student? Whether there is any potent argument to be set forth against it or not, *he goes*, and in swarms, and the seductive music of the military bands and the "damen capellen" (lady orchestras) is sufficient to engross their attention until very late—or rather early—hours, sometimes. I don't wonder that German gentlemen spend most of their life in obtaining their education!

But I must not devote too much space to this aspect of medical study, or it may be thought that I have allowed myself to participate in such frivolities, or that that is the chief aim of the fraternity here. Let me not convey any such impression. A vast deal of good hard work is done here, both by the students (especially the Americans, who do not so readily adopt the easy and slow-going methods of the Germans) and by the teachers, who stand as forcible exemplifications of the reward to be derived from merit and industry.

The distinction of a professorship here means much more than it does in our country. Professor Dittel, who is now 73 years of age, received his professorship only about eleven years ago. The students are all much older than our medical students are; they hardly graduate before reaching the thirties, and are asssistants at the clinics, or are "private docents" until well along toward middle life. So that it is apparent that the young man of this land is about ready to shuffle off the mortal coil by the time he has attained the aim of his ambition—a professorship in the University. Dr. Ernst Finger is about the youngest of those who are to be numbered amongst the prominent; and his success is as deserving as it is pronounced, considering the exceedingly creditable work he has done in his special field—genito-urinary. He appears to be about 38 years of age; he has written two books, one on gonorrhœa, this probably being the best work extant on the subject. Praise of the course given by him is universal, his methods of diagnosis and treatment of urethral affections being both rational and scientific, the accuracy of which is daily confirmed by the excellent results obtained at his clinic. The material appearing there daily is large, varied and always interesting, especially so as its lessons are interpreted in such an instructive and lucid manner. The number of cases of spermatorrhœa, demonstrated to be really such by the microscope, would surprise some of those who claim that this is, for the most part, a disease of the imagination. Cases of impotence of various degrees of severity are here not treated mainly with—incredulity and advices, which together seem to be the "sheet-anchor" of some practitioners after whom I have read, A lesion clearly demonstrable for these and other obscure sexual disorders, is usually discovered by the refined methods of diagnosis employed at this clinic. And it is for this part of his work that Dr. Finger deserves most credit.

It seems to be his aim to impart to his hearers appreciation of these diseases in their physiological relation, such an understanding furnishing proper basis for the great *primary object* of the would-be healer, that is, a *definite* and *accurate diagnosis* of the trouble. The diagnosis is the important part of the problem; after that is made clear, proper therapeutic measures are arrived at. (N. B. Whether they will always suceeed in curing the case, I will not at present presume to say, as I bear in mind the wonted perversity of genito-urinary diseases in general; but the success which Dr. Finger obtains in the relief of old, chronic urethral cases, is soon made apparent to those who follow them in the clinic). This course seems to be a favorite one with the Americans, nearly the whole of the present class being made up from our colony.

I notice that my letter is becoming prolonged to an unseeming degree, and I have not conformed to the orthodox regulations established by "foreign correspondents," of adhering strictly to the soberer topics of advances in medicine, discussions of societies, etc. Perhaps I shall do better next time.—*Dr. Bransford Lewis in the Medical Herald.*

---

IODOFORM IN CHRONIC EYE DISEASES.—G. Walter Barr, M. D., in *Therapeutic Gazette*, April, 1891:

Uses it in the form of ointment, one to five grains iodoform to the ounce of vaseline, urging the necessity of pure articles of each. Used it in one hundred cases of trachoma, mostly of very long standing; thirty cases of pannus, and a dozen cases of corneal ulcer.

He varies the strength of the ointment to suit the individual case, with excellent results.

In pannus, apply ointment often enough to keep cornea coated with it. In granular lids vary the strength sufficient to keep up the desired amount of stimulation—usually requires four grains ointment applied twice daily. The granulations quickly subside under this treatment.

In indolent corneal ulcers, the effect is as marked. Ulcers occasionally require touching with cupric sulphate.

---

SIMPLE TREATMENT OF INGROWN TOENAIL.—The Pittsburg *Medical Review*, February, 1891, cites Dr. Puerckhauer (from the *Munchner Med. Wochenschrift*) as recommending a simple and at the same time competent treatment for ingrown toenail.

A forty per cent. solution of potassa is applied warm to the portion of the nail to be removed. After a few seconds the uppermost layer of the nail will be so soft that it can be scraped off with a piece of sharp-edged glass; the next layer is then moistened with the same solution, and scraped off; this must be repeated until the remaining portion is as thin as a sheet of paper, when it is seized with a pincette and lifted from the underlying soft parts and severed from the other half. The operation does not require more than half an hour's time, is painless and bloodless, while the patient is delivered fromhis suffering without being disabled even for an hour.—*American Prac. and News.*

ON THE ACTION OF ATROPINE IN DISEASES OF THE HEART.—Dr. Cardarelli has experimented with atropine in sixty-five cases of various forms of functional cardiac disease, measuring the pulse with the chromograph of Verdin, and the arterial pressure with the sphygmo-monometer of Basch. His conclusions, which appear to be based on a most careful study, are published in La France Medicale, Jan. 23, 1891:

1. Atropine, in doses of 1-120 to 1-32 grain, given hypodermically in man, manifests itself first in its action on the heart.

2. The action of atropine on the heart consists in the overcoming to a greater or less degree of the inhibitory influence of the vagus nerve.

3. As a consequence of this paralyzing action on the vagus, there is constant acceleration of the cardiac rhythm, which may be in certain cases accompanied by a slight transitory slowing.

4. Arterial pressure is reduced under the influence of atropine in direct proportion to the acceleration of the rhythm.

The author concludes with the statement that, when a clinician cannot use atropine in slight forms of irritation of the pneumogastric, in which there is no slowing of the pulse, it will be nevertheless a great error not to prescribe atropine in cases where a permanently slow pulse is accompanied by epileptiform vertigo, and above all, by syncope.—*Therapeutic Gazette.*

---

ALCOHOL AND DIGESTION.—From experiments made on himself by Dr. Eichenberg, some further knowledge of the effect of alcohol on digestion is obtained, which contrasts strongly with the teetotal lecturer's experiment showing how digestion in a glass vessel is retarded by alcohol. Dr. Eichenberg found that a strong dose of alcohol (for example brandy) shortens the time that food in general, whether animal or vegetable, or a mixture, remains in the stomach by more than half an hour. A similar but not quite so marked an effect is produced by a small dose of diluted hydrochloric acid or mustard. Pepper and condurango diminish the time the food remains in the stomach by about a quarter of an hour. Beer and an infusion of rhubarb had no effect.—*American Prac. and News.*

